# Heritability of symbiont density reveals distinct regulatory mechanisms in a tripartite symbiosis

**DOI:** 10.1002/ece3.2005

**Published:** 2016-02-26

**Authors:** Jasmine F. Parkinson, Bruno Gobin, William O. H. Hughes

**Affiliations:** ^1^ School of Life SciencesUniversity of SussexBrightonBN1 9QGU.K.; ^2^ PCS‐Ornamental Plant ResearchSchaessestraat 18Destelbergen9070Belgium

**Keywords:** Endosymbionts, Hemiptera, infection density, mealybug, *Planococcus citri*, Proteobacteria

## Abstract

Beneficial eukaryotic–bacterial partnerships are integral to animal and plant evolution. Understanding the density regulation mechanisms behind bacterial symbiosis is essential to elucidating the functional balance between hosts and symbionts. Citrus mealybugs, *Planococcus citri* (Risso), present an excellent model system for investigating the mechanisms of symbiont density regulation. They contain two obligate nutritional symbionts, *Moranella endobia*, which resides inside *Tremblaya princeps*, which has been maternally transmitted for 100–200 million years. We investigate whether host genotype may influence symbiont density by crossing mealybugs from two inbred laboratory‐reared populations that differ substantially in their symbiont density to create hybrids. The density of the *M. endobia* symbiont in the hybrid hosts matched that of the maternal parent population, in keeping with density being determined either by the symbiont or the maternal genotype. However, the density of the *T. princeps* symbiont was influenced by the paternal host genotype. The greater dependency of *T. princeps* on its host may be due to its highly reduced genome. The decoupling of *T. princeps* and *M. endobia* densities, in spite of their intimate association, suggests that distinct regulatory mechanisms can be at work in symbiotic partnerships, even when they are obligate and mutualistic.

## Introduction

Symbiotic associations are extremely widespread in nature, and beneficial eukaryotic–bacterial partnerships have shaped the very foundations of plant and animal evolution (Schwartz and Dayhoff 1978). Symbiosis creates an overlap of selective interests between partners, which will increase with the degree to which symbiont transmission is vertical rather than horizontal, and will be strongest in the hosts that vertically transmit the symbiont (usually females). However, even mutualistic symbiotic associations are inherently selfish, with benefits given only so long as they are reciprocated and, as well as selection for cooperation, there is also selection pressure to cheat and exploit the partnership (Bennett and Moran 2015). As they coevolve, hosts will be selected to increase their own fecundity, with or without symbionts, whereas symbionts will be selected to maximize their transmission to new hosts, while simultaneously outcompeting other strains and species of symbiont for the limited resources provided by the host (Frank 1996).

This conflict of selective interests between hosts and symbionts can in part be resolved by vertical transmission of the symbionts, and consequent dependency of the symbiont upon the host. Guaranteed vertical transmission to the next generation relaxes the selection pressure for horizontal transmission, leads to genetic homogeneity within hosts, and thus favors decreased virulence of symbionts (Frank 1996; Smith 2007). Evidence for this tendency to transition to avirulence and homogeneity within hosts can be observed in organelles (Birky et al. 1983), and the *Uroleucon ambrosiae* symbionts of aphids (Funk et al. 2000). Symbiont dependency upon the host increases following symbiont genome reduction – a common result of the symbiont lifestyle (Moran and Bennett 2014; Bennett and Moran 2015).

Even in the case of vertically transmitted symbionts, strict regulation of symbiont density within the host is essential for the efficient functioning of the partnership (Falkowski et al. 1993; Rio et al. 2006; Wilkinson et al. 2007; Cunning and Baker 2014; Laughton et al. 2014). Too few symbiont cells will cause a deficiency of gene products for the host and inefficient vertical transmission for the symbiont, while too many cells will incur some cost to the host without a proportionate benefit. As accommodating a symbiont, even when it is beneficial, will always incur some cost to the host in terms of energy or resources (Bronstein 2001), an excess of symbionts could also be metabolically demanding to the host. Costs to the host could lead to long‐term costs to the symbionts through reduced host fecundity and hence reduced vertical transmission. In terms of host fitness, the “optimum” within‐host symbiont density will be complex and dynamic, being unlikely to be constant throughout the lifecycle of the host, or in every environmental situation that the host encounters, but will instead change depending on context, and be subject to multiple, possibly conflicting, selection pressures and host requirements. Facultatively manipulating symbiont density may prove to be costly to the host. Additionally, the symbiont will be selected to maintain at minimum the threshold density required to ensure vertical transmission, which may in itself vary throughout the life of the host. There may then be selection on both host and symbiont to maintain a compromised symbiont density across environmental and physiological conditions (Rio et al. 2006; Kono et al. 2008; Laughton et al. 2014).

Regulation of symbiont density can occur via the host or the symbiont. Symbionts may change their density by varying their replication rate to maintain or increase their density, whereas hosts can control symbionts using several mechanisms. Depending upon the method of transmission, a screening process can prevent unwanted symbionts from entering the host (Nyholm and McFall‐Ngai 2004). Antimicrobial peptides, in some cases symbiont‐specific, can be deployed (Balmand et al. 2011; Hooper et al. 2012). Superfluous bacteria can in some cases be simply evicted (Ruby and Asato 1993; Dimond and Carrington 2008). Nutrient acquisition by the host is positively correlated with symbiont density in pea aphids and some corals, which may be a limiting factor in the proliferation of symbionts, and facilitates homeostasis between two sympatric teste fly symbionts (Falkowski et al. 1993; Muller‐Parker et al. 1994; Wilkinson et al. 2007; Snyder et al. 2010). Regulatory mechanisms may be linked, rather than acting in isolation, for example, the rates of degradation and expulsion of zooxanthellae by the coral, *Stylophora pistillata*, are both triggered by starvation of the host (Titlyanov et al. 2000).

Immune mechanisms in some host species still provide a sophisticated form of symbiont density control (Hinde 1971; Falkowski et al. 1993; Bennett and Moran 2015). Indeed, maintaining a symbiont requires that the host amend its approach to dealing with internal bacteria, and suppress or adjust its immune responses (Wang et al. 2009; McFall‐Ngai et al. 2010; Ratzka et al. 2013). For example, the pea aphid *Acyrthosiphon pisum*, has lost genes involved in the IMD immune pathway (Gerardo et al. 2010, The International Aphid Genomics Consortium, 2010).

Citrus mealybugs, *Planococcus citri* (Risso), are an intriguing and potentially powerful model system for investigating the roles of host and symbiont in regulating symbiont density. Citrus mealybugs contain two maternally, vertically transmitted obligate nutritional symbionts, a *β*‐proteobacterium, *Tremblaya princeps*, and a *γ*‐proteobacterium, *Moranella endobia*, which reside in bacteriocytes in the bacteriome organ surrounding the host gut (Thao et al. 2002). These two symbionts have coevolved intimately, with *M. endobia* actually residing inside *T. princeps*, which was first acquired by the Pseudococcidae 100–200 million years ago (Baumann et al. 2002; Thao et al. 2002; Husnik et al. 2013).

Both symbionts have reduced genomes (Baumann et al. 2002; Husnik et al. 2013), which could potentially compromise their ability to self‐regulate their density within the host. Genome reduction is a common Muller's Ratchet‐type consequence of the relieved natural selection pressures experienced by intracellular bacteria (McCutcheon and Moran 2012; Moran and Bennett 2014). *T. princeps* holds one of the smallest bacterial genomes known to science, at just under 139 kb (Husnik et al. 2013), while *M. endobia* carries a larger, yet still reduced, genome of 538 kb (McCutcheon and von Dohlen 2011). It is hypothesized that the dramatic gene loss experienced by *T. princeps* is partly due to it harboring its own symbiont which can compensate for loss of genetic function (Husnik et al. 2013).

*Tremblaya princeps* relies on both the mealybug host and *M. endobia* to counteract its loss of genes and their functions, which could render it dependent on these partners to regulate its density (Lopez‐Madrigal et al. 2011; McCutcheon and von Dohlen 2011; Husnik et al. 2013; Sloan et al. 2014). For example, genes involved in the construction of cell wall components are found horizontally transferred from other bacterial species into the mealybug genome and are highly expressed in the bacteriocytes where *T. princeps* resides (Husnik et al. 2013), and translation‐related genes no longer present in *T. princeps* are expressed in *M. endobia* (McCutcheon and von Dohlen 2011).

There is some evidence for genotypic differences symbiont density within *P. citri*. Citrus mealybug populations have been found to differ in the density of both of their bacterial symbionts by over sixfold, even when cultured under standard laboratory conditions (JFP, BG & WOHH, unpubl. data). The consistency of differences in symbiont density between mealybug populations supports the case for genotypic variation in the propensity to harbor a high or low symbiont density in citrus mealybugs. However, it is not clear whether the differences between populations are caused by the genotype or epigenetics of the host or of the symbiont. In this study, we disentangle the effects of host genome from symbiont genome by crossing mealybugs from two inbred laboratory‐reared mealybug populations that differ substantially in their symbiont density in order to create F_1_ hybrid daughters. These hybrid mealybugs host the symbionts from their maternal population because symbiont transmission is entirely maternal (Thao et al. 2002), but will have a genome that is derived from both paternal and maternal parents. Any significant deviation in symbiont density from the maternal population would therefore be attributable to the paternal genotype, and indicative of host genotype influencing symbiont density. Alternatively, a nonsignificant deviation in symbiont density from the maternal population would indicate that symbiont density is determined only by symbiont genotype (or maternally specific genotypic effects such as via imprinting).

## Methods

Two mealybug populations (A and B) were used which had been obtained from commercial greenhouses in Belgium and cultured in darkness at 25°C and 20% RH on white organic potato sprouts for 8 months (approximately eight generations). These populations had been found previously to differ approximately twofold in the densities of both the *M. endobia* and *T. princeps* symbionts (Parkinson et al. unpublished). Newly emerged adult females from these populations were separated from their populations of origin and maintained on potato sprouts for 5 days. Any females which commenced oviposition in this time period were discarded (ca. 20% of females) to ensure virginity. Adult males from the other population were then placed with the females for 48 h to allow for mating (males from Population A were placed with females from Population B and vice versa). This hybridization process created two F1 generation hybrid populations: A♀B♂ and A♂B♀. When each female commenced oviposition, she was placed on an individual potato to lay eggs in isolation. The F1 hybrid offspring from each female were allowed to hatch and mature on these isolated potatoes, with all male offspring being removed to ensure the virginity of their sisters. F1 females were allowed to grow to maturity (~ 30 days posthatching).

### Symbiont quantification

Newly emerged adult females from Populations A (*n* = 39) and B (*n* = 40) and the hybrid populations A♀B♂ and A♂B♀ (20 offspring per mother, *n* = 28 mothers for A♀B♂, *n* = 29 mothers for A♂B♀) were crushed individually in 100 *μ*L 5% chelex solution, heated to 99°C for 15 min and centrifuged at 2326 g for 20 min. The supernatant was pipetted off and diluted 1:10 with molecular grade water for use in qPCR reactions. DNA from multiple offspring was pooled to create a single DNA sample per mother.

Symbiont infection intensity was quantified by measuring gene copy number using qPCR with the comparative C_T_ method, using the host *28S* gene to control for DNA quantity (Schmittgen and Livak 2008), as per (Parkinson et al. 2014). Primers and probes for the *P. citri* control gene, 28S rDNA and *T. princeps GroEL* gene were designed using PRIMER3 software (Whitehead Institute for Biomedical Research, Cambridge, MA) and analyzed using NetPrimer software (Primer Biosoft International, Palo Alto, CA). Primers and probes for *M. endobia 16S* and *23S* rDNA were designed using Primer Express v.3.0 software (Life Technologies, Foster City, CA) (Table 1). To ensure that only a single PCR product would be amplified for *M. endobia*, the forward primer for *M. endobia* was checked against the *M. endobia* complete genome (Accession number CP003881.1), which was isolated from the citrus mealybug PCVAL strain, and found to match at only a single site (López‐Madrigal et al. 2013). The forward primer also only matched a single site for the *M. endobia* complete genome (Accession number CP002243.1), which was isolated from the citrus mealybug PCIT strain (McCutcheon and von Dohlen 2011). To ensure that only a single PCR product would be amplified for *P. citri*, the forward primer for *P. citri* was checked against *28s rDNA* GenBank sequences (Accession numbers GU134660.1, JF714181**.**1, JQ651165.1, JQ651169.1, JQ651170.1, JQ651171.1, JQ651362.1, JQ651363.1, JQ651364.1, JQ651365.1) and found to match at only a single site (Malausa et al. 2011; Beltrà et al. 2012; Sethusa et al. 2013). The *28S rDNA* gene has been used for several phylogenetic studies in mealybugs, with being present as a single copy in citrus mealybugs (Downie and Gullan 2004; Hardy et al. 2008). A total of 10‐*μ*L reaction volumes were used for qPCR in a StepOnePlus^™^ Real‐Time PCR System (Applied Biosystems Foster City, California, United States), with 150 nmol/L of each primer, 50 nmol/L of probe, and 1× of ABI Taqman Universal Master Mix II with UNG (Life Technologies). The cycle was 50°C for 2 min, 95°C for 10 min, followed by 40 cycles of 95°C for 15 sec and the annealing temperature (collection step) for 1 min. An annealing temperature of 64°C was used for *P. citri* and *M. endobia* reactions, and 60°C was used for *T. princeps* reactions.

**Table 1:** qPCR primers and probes used in this study for *Planococcus citri* host control, *β*‐proteobacterial symbiont, *Tremblaya princeps*, and *γ*‐proteobacteria symbiont, *Moranella endobia*

Target organism	Target gene	Oligo name	Function	Fluorescence^a^	Oligo sequence 5'‐3'	Product size (bp)
*P. citri*	28S rDNA [AY179451.1]	*PcitriF*	Forward primer	–	TCCGAGGAGACGTGTAAAAGTTC	56
		*PcitriR*	Reverse primer	–	CCTAGCCGCCGAAACGA	
		*PcitriP*	Probe	6FAM	ACGGCGCGTGTCGA	
*T. princeps*	*GroEL* [AF476091]	*TprincepsF*	Forward primer	–	TCCAAGGCTAAATACCCACA	155
		*TprincepsR*	Reverse primer	–	ATACAAAAGGTACGCCGTCA	
		*TprincepsP*	Probe	6FAM	CGCGCATACGAACAGTCGGA	
*M. endobia*	*16S* and *23S* rDNA [AF476107.1]	*MendobiaF*	Forward primer	–	GAGCACCTGTTTTGCAAGCA	64
		*MendobiaR*	Reverse primer	–	CCCCTAGAGTTGTGGAGCTAAGC	
		*MendobiaP*	Probe	6FAM	AGTCAGCGGTTCGATC	

a 6FAM, 6‐fluorescein amidite 5' dye.

The densities of *T. princeps* and *M. endobia* in individual mealybugs were determined by comparing symbiont gene copy number against the *P. citri* host control gene, using the comparative C_T_ method, which standardizes for differences in tissue quantities (Schmittgen and Livak 2008; Crotti et al. 2012). All samples were run in triplicate and nonconcordant replicates and samples were rerun or excluded. The C_T_ values of all three target genes were measured for each mealybug. Then the difference in C_T_ value between the symbiont genes and the host control gene for each mealybug were calculated and expressed as fold differences in the symbiont genes relative to the host genes by 2^−(symbiont CT − host CT)^.

### Statistical analysis

Analysis was conducted by converting relative ∆C_T_ values into host–symbiont ratios. Symbiont densities in the different populations were analyzed using a generalized linear model with a gamma distribution and log‐link function and the Likelihood ratio *χ* ^2^ statistic. The sequential Bonferroni correction to the Wald test was used for pairwise comparisons of populations. Data for *T. princeps* and *M. endobia* were analyzed separately. Differences in extraction and quantification efficacies for the two symbionts mean that the quantities cannot be compared between the symbionts.

## Results

**Figure 1:**
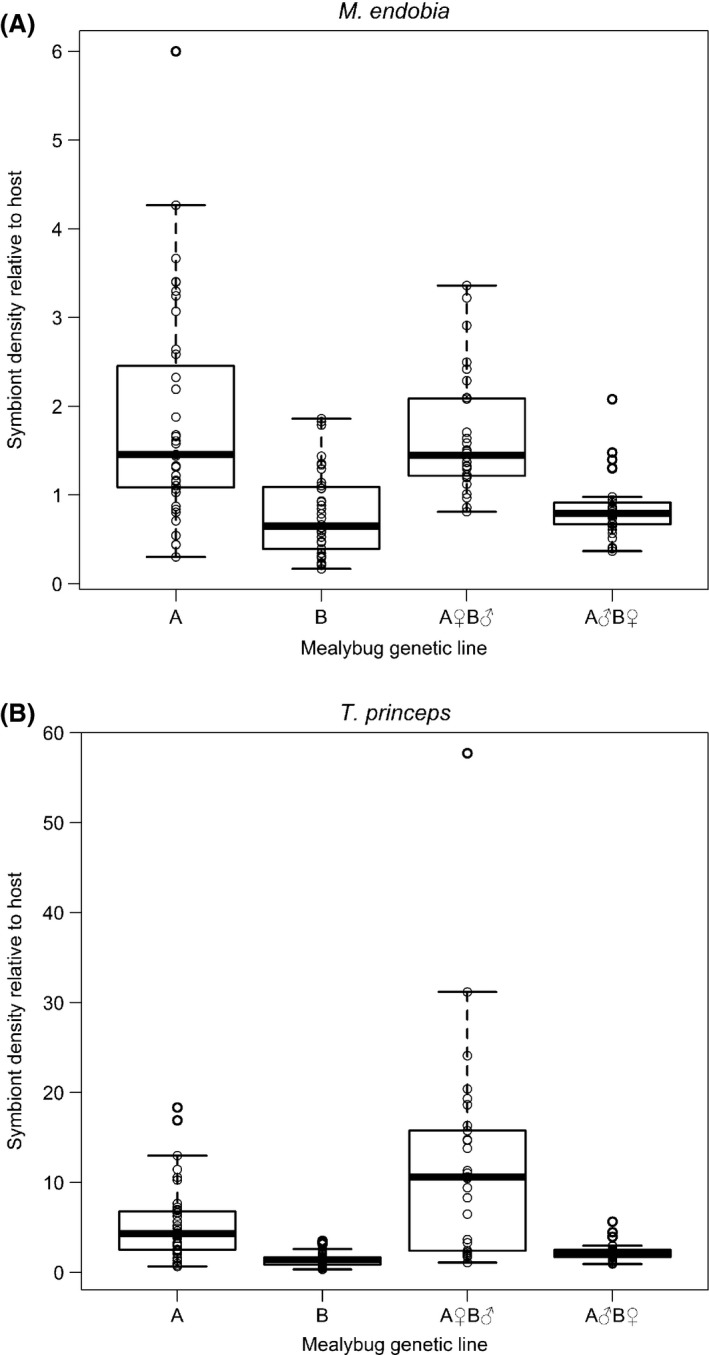
The mean, quartiles, 95th percentiles and individual data points of the densities (relative to the host control gene) of the (A) *M. endobia* and (B) *T. princeps* bacterial symbionts in adult citrus female mealybugs from parental populations A and B, and the hybrid offspring populations A♀B♂ and B♀A♂. Symbiont density was measured using qPCR, calculated as relative to *P. citri* host control gene using the comparative C_T_ method.

The qPCR data gave us the relative infection intensity of the two bacterial symbionts in the two parent populations of mealybugs and their hybrid daughters (see Supplementary Information). The relative infection intensity of the *M. endobia* symbiont differed significantly between the mealybug populations (*χ* ^2^ = 56.4, df = 3, *P* < 0.001). Population B had on average 58% fewer *M. endobia* cells per host cell than Population A (Fig. 1A). Pairwise comparisons reveal that the F_1_ hybrid populations differed significantly from their paternal populations (*P* < 0.001 in both instances), but not their maternal population (*P* = 0.892 for A♀B♂ and *P* = 0.141 for A♂B♀).

The same pattern did not follow for the *T. princeps* symbiont (Fig. 1B). Symbiont density again differed significantly between mealybug populations (*χ* ^2^ = 85.3, df = 3, *P* < 0.001), and Population B had on average 71% fewer *T. princeps* cells per host cell than Population A. However, pairwise comparisons revealed that both F_1_ hybrid populations differed significantly from not only their paternal populations (*P* < 0.001 in both instances) but also both their maternal populations (*P* = 0.010 for A♀B♂ and *P* < 0.001 for A♂B♀). Population A♀B♂ had a *T. princeps* density that was higher than either of its parent populations (185% greater than that of Population A), while Population A♂B♀ had a *T. princeps* density intermediate between those of its parent populations (51% of that of Population A; Fig. 1B).

## Discussion

In order to separate the effects of bacterial‐derived versus host‐derived regulation of symbiont density, we crossed two laboratory strains of citrus mealybug with consistently different infection intensities of the *T. princeps* and *M. endobia* symbionts to create two new hybrid strains. *M. endobia* densities in adult females from these hybrid strains were not significantly different from those of the maternal populations, indicating that *M. endobia* density was not affected by host paternal genotype. However, *T. princeps* densities in adult females from these hybrid strains were significantly higher than from those of their maternal populations, indicating that the paternal host genotype influenced the density of the symbiont, possibly in a nonadditive way as the hybrid strain A♀B♂ had a *T. princeps* density that was higher than either of the parental populations. This may also have been a result of heterosis of the host genome, which may have enabled the host to harbor more *T. princeps* cells. Despite this, the hybrid strains still held a *T. princeps* density that was more similar to the maternal than the paternal line, so *T. princeps* may still to some degree control over its density.

*Planococcus citri* holds a logistical advantage for regulating its symbionts' densities. *T. princeps* and *M. endobia* reside in specialized bacteriocytes which compose the bacteriome organ surrounding the gut of the host, a prime location for nutritional symbionts to function (Thao et al. 2002). Cordoning symbionts into a single location also eases organized density control and bacteriocytes often express high levels of antimicrobial peptides, such as observed in the rice weevil, *Sitophilus oryzae* (Login et al. 2011). *Bemisia tabaci* whiteflies are less capable of effectively regulating symbionts that are situated outside of their bacteriocytes (Su et al. 2014).

The decoupling of *T. princeps* and *M. endobia* densities suggests that, despite their intimate evolutionary association, distinct regulatory mechanisms are at work for the two symbionts. Decoupling of the two symbionts has been observed in adult male mealybugs, who lose *M. endobia* at a faster rate than *T. princeps* as they approach their aposymbiotic stage (Kono et al. 2008). Differential regulation mechanisms for obligate versus facultative symbiont density have also been found in the pea aphid, revealed by varying dietary nitrogen levels (Wilkinson et al. 2007), reflecting the distinct relationships that aphids share with different types of symbiont. However, *T. princeps* and *M. endobia* are both obligate nutritional mutualists and, moreover *M. endobia* resides inside *T. princeps*, so their inconsistent responses to hybridization are surprising.

The nested relationship of *M. endobia* inside *T. princeps* and their discrepancies in genome size may account for their different density regulatory mechanisms. *T. princeps* has a dramatically reduced genome, one of the smallest known to science with only 120 protein‐coding genes, and relies on *M. endobia* and the host for much of its function (Husnik et al. 2013). It is argued that in terms of gene number and genome size, *T. princeps* is more similar to an organelle than a symbiont (McCutcheon and Moran 2012; Husnik et al. 2013). It could be argued that such dependence and efficient vertical transmission will mean that *T. princeps* may thus behave as a part of *P. citri*, rather than a separate organism within *P. citri* with its own conflicting evolutionary interests. However, even intragenome conflict can occur, and the fitness requirements of one individual in a symbiotic relationship is unlikely to align flush with that of its partner (Eberhard 1980; Herre et al. 1999). Even organelles can still conflict with their hosts, for example the CMS (cytoplasmic male sterility) induced by mitochondria in some plant species (Chase 2007). Uniparental transmission benefits hosts by preventing competition between unrelated organelles, but deems one of the sexes to be an evolutionary dead end for the organelles (Law and Hutson 1992; Hurst 1995).

*Tremblaya princeps* has lost functional genes for bacterial translational release factors, aminoacyl‐tRNA synthetases, ribosome recycling factor, elongation factor EF‐Ts, and peptide deformylase (McCutcheon and von Dohlen 2011). It is common for symbionts to lose genes associated with cell wall structure, for example, *T. princeps* lacks cell envelope‐related genes and relies on its host for the creation of a cell membrane (McCutcheon and von Dohlen 2011; McCutcheon and Moran 2012; Husnik et al. 2013). It could therefore be the case that the larger and more functionally complete genome of *M. endobia* gives it more control of its own regulation, than *T. princeps*. However, the expression of *murABCDEF* and *mltD/amiD* genes in the host genome is believed to control the cell wall stability and lysis of *M. endobia*, so even this symbiont may still be partially influenced by its host's genotype (McCutcheon and von Dohlen 2011; Husnik et al. 2013, 2013; Koga et al. 2013).

In summary, the decoupling of *M. endobia* and *T. princeps* densities following crossing of mealybug lines with different symbiont infection intensities reveals that even nested intracellular symbionts can have different regulatory mechanisms. *T. princeps* provides an example of how the defined boundary between organism and organelle can be blurred, and, despite their antiquity, it may be more appropriate to consider organelles as part of the same evolutionary spectrum as symbionts rather than a discrete functional category (McCutcheon and Keeling 2014). Understanding the density regulatory mechanisms behind bacterial symbiosis will be essential to understanding the functional balance between hosts and symbionts, and how they have evolved to overcome their conflict of interests.

## Conflict of Interest

None declared.

## Supporting information


**Table S1** The mean CT values of the host control *P. citri* 28S gene, target *M. endobia* 16S and 23S rDNA and target *T. princeps GroEL* gene from individual adult female P.
